# Self-control or social control - what determines sleep hygiene in bed-sharing couples?

**DOI:** 10.5935/1984-0063.20200095

**Published:** 2021

**Authors:** Henning Johannes Drews, Robert Göder, Panagiotis Mitkidis

**Affiliations:** 1 Norwegian University of Science and Technology (NTNU), Department of Mental Health - Trondheim - Trøndelag - Norway.; 2 Christian-Albrechts-University Kiel, Department of Psychiatry and Psychotherapy - Kiel - Schleswig-Holstein - Germany.; 3 Aarhus University, Department of Management - Aarhus - Midtjylland - Denmark.; 4 Duke University, Center for Advanced Hindsight, Social Science Research Institute - Durham - North Carolina - United States.

**Keywords:** Sleep Hygiene, Co-sleep, Bed-sharing, Self-control, Social Control, CBTI

## Abstract

**Objectives:**

To investigate intimate partners’ impact on sleep hygiene with focus on the temporal dimension and differential predictors of sleep hygiene in co-sleepers and individual sleepers.

**Material and Methods:**

Habitual co-sleepers and individual sleepers (n=102) completed a cross-sectional, self-report, in-lab, digital survey on sleep hygiene, habitual sleeping arrangement, self-control, depressiveness, and sociodemographic parameters.

**Results:**

The relationship between sleeping arrangement and sleep hygiene in co-sleepers was time-dependent with an initial steep incline and a subsequent plateau at approximately one year of co-sleeping routine. Co-sleepers with more than one year of unaltered sleeping arrangement had significantly better sleep hygiene than co-sleepers with less than one-year or individual sleepers. More than one-year continuity of the sleeping arrangement moreover robustly predicted sleep hygiene in co-sleepers whereas self-control was the dominant predictor in individual sleepers.

**Conclusion:**

Amongst others, our findings support the idea that insomnia treatment could be improved by becoming sensitive to the habitual sleeping arrangement.

## INTRODUCTION

There is growing interest in the effects of intimate relationships on sleep^1-4^. However, much of the hitherto research has focused on personal factors (e.g., personality traits, attachment styles) or relational functioning (e.g., marital satisfaction, inter-partner conflict; for review see Rogojanski et al. (2013)^1^. Two potentially relevant fields are understudied: first, the temporal dimension of a relationship (i.e., relationship duration and routine of co-sleeping). Here, Troxel et al. (2010)^5^ reported a beneficial effect of being in a relationship on sleep, only for long-term relationships. Second, the partner’s influence on behavior that directly promotes good sleep (i.e., sleep hygiene). This is of particular interest since sleep hygiene is an important part of cognitive behavioral therapy for insomnia (CBTI) and including the intimate (bed) partner into CBTI has been suggested to potentially improve CBTI^1^.

In this context, a social-control hypothesis of sleep behavior has been put forward. It postulates that partners tend to monitor and try to influence their partners’ health behavior by reminding and requesting engagement in a healthy lifestyle. While this has been shown for nicotine consumption and physical exercise^6^, evidence for a partner’s impact on sleep-related health behavior (i.e., sleep hygiene) is still missing^1^. The concept of social control through a partner resonates with the concept of self-control, i.e., “the ability to override or change one’s inner responses, as well as to interrupt undesired behavioral tendencies”^7^, which has similarly and independently been argued to determine health behavior^7^. However, neither self-control nor the presence of a partner has sufficiently been tested regarding their relevance for sleep hygiene.

The present study seeks to investigate the specific effects of a bed partner on sleep hygiene by comparing habitual bed-sharers to individual sleepers.

We hypothesize that: i) bed-sharers have a better sleep hygiene than individual sleepers with a time-dependent, biphasic distribution (i.e., an adaptational phase, during which sleep hygiene improves, followed by a plateau phase); ii) following the idea of social and self-control we hypothesize that sleep hygiene in bed-sharers is governed by a routinely present social factor, that is, a co-sleep duration that has exceeded the adaptational phase, whereas self-control is the most important predictor of individual sleepers’ sleep hygiene.

The significance of testing these hypotheses lies - amongst others - in potentially providing starting points to improve CBTI by making it sensitive to the habitual sleeping arrangement.

## MATERIAL AND METHODS

### Study design and procedure

The present study draws on data of an observational, cross sectional, monocenter study consisting of a self-report, in-lab, digital survey that was derived from standardized and validated measures that were filled in at a single uninterrupted session (taking approximately 30-45 minutes). In some cases, adaptations in the Likert scales were done to align questionnaires amongst each other (please note that this mainly concerns turning 5-point Likert scales into 7-point Likert scales, which has been shown not to alter data characteristics)^8^. The questionnaire was administered in English via Qualtrics survey software. Each participant received approximately $15 in local currency (DKK 100.00) for participating in the study. Prior to study initiation the local lab’s ethical review board approved the project (ID 0253). Written informed consent was obtained. All data were fully anonymized.

### Sampling and sample characteristics

Participants were recruited by convenience sampling from an unpublished larger study (n=189) concerning decision-making styles which had used subjects from the Sona subject pool at Aarhus University. Inclusion criteria were English language proficiency and having an unambiguous sleeping arrangement (i.e., bed-sharing or individual sleep with no roommates; n=102). This procedure resulted in n=40 habitual individual sleepers (15 male) and n=62 habitual co-sleepers (21 male; median relationship duration: 30 months (IQR: 17 to 38). Mean (±SD) age was 26.3±8.2 years. National backgrounds were Danish (n=42), from other European countries (n=38), North American (n=4), South American (n=9), Asian (n=8), and other (n=1). Details are given in Table 1.

**Table 1:** Sample characteristics.

	Male	Female	p-value
**Gender**			
Overall n=102	36	66	
Individual sleepers n=40	15	25	0.871
Co-sleepers n=62	21	41	
	**Mean**	**SD**	**p-value**
**Age [years]**			
Overall	26.29	8.16	
Individual sleepers	26.93	10.38	0.397
Co-sleepers	25.89	6.39	
**Self-control [score]**			
Overall	50.62	12.16	
Individual sleepers	51.80	13.99	0.458
Co-sleepers	49.85	10.87	
**Depressiveness [score]**			
Overall	4.37	2.11	
Individual sleepers	4.33	2.37	0.538
Co-sleepers	4.40	1.94	
**Sleep hygiene [Score]**			
Overall	51.59	13.04	
Individual sleepers	49.02	13.39	0.116
Co-sleepers	53.24	12.64	
**Continuity of sleeping arrangement [months]**			
Overall	36.94	60.27	
Individual sleepers	53.36	84.15	0.880
Co-sleepers	26.35	34.59	
**Sleep quality [score]**			
Overall	3.31	1.49	
Individual sleepers	3.40	1.55	0.604
Co-sleepers	3.26	1.46	

**Notes:** “Continuity of sleeping arrangement” refers to the time span prior to study participation during which the participants slept in the same sleeping arrangement (co-sleeping or individual sleep). The *p*-values represent group comparisons individual sleep vs co-sleep. Tests: Fishers exact test for count data (gender distribution), two-sided, unpaired t-tests (self-control and sleep quality), two-sided, Wilcoxon rank sum tests (age, depressiveness, continuity of sleeping arrangement, and sleep hygiene).

### Measures

#### Sleep hygiene

The sleep hygiene index (SHI)^9^ is a 13 items instrument that assesses classical sleep hygiene content (e.g., bed-time regularity, sleeping environment, rumination, and substance use before going to bed). The SHI has been reported to correlate highly with sleep quality^9^.

For the present study we modified the five-point Likert scale to a seven-point Likert scale (ranging from 1 to 7 = min 13, max 91) and inverted the orientation with now higher values indicating better sleep hygiene. To ensure that the changes would not affect the validity of the scale, we compared it to the subjective sleep quality question of the Pittsburgh sleep quality index (PSQI)^10^ which we also modified to a seven-point Likert scale (1 to 7, lower values indicating higher sleep quality). As expected, there was a very significant correlation between subjective sleep quality and our modified SHI (r=-0.31, *p*=0.001, n=102, Spearman correlations).

#### Sleeping arrangement, self-control, and depressiveness

In addition to assessing the habitual sleeping arrangement, we asked for unchanged continuity of the respective sleeping arrangement prior to our study (“how long has the (sleeping) situation been like this?”). Self-control was assessed by using the scale by Tangney et al. (2004)^7^ that has been shown to correlate with a variety of health behaviors and societal functioning^7^. The self-control scale is a 13-item self-rated questionnaire in which agreement to several statements is expressed on a Likert scale. Here we used a seven-point scale (1=“not at all”, 7=“very much”). Exemplary statements are: “people would say that I have iron self-discipline.”, or “I often act without thinking through all the alternatives”. Of the 13 items 8 are reversed so that higher values indicate higher levels of self-control.

Additionally, since sleep, health behavior, and self-control have been reported to be closely related to depressiveness^7,11,12^, we included depressiveness into our analysis as a potential confounder variable. Depressiveness was assessed via an efficient two-question method (asking for depressed mood and lack of interest/pleasure) for which a high sensitivity and good specificity has been reported^13^. The sum scale ranged from 2 to 10 with higher values indicating higher levels of depressiveness.

### Statistical analysis

After testing for normal distribution (Shapiro-Wilk tests), we compared habitual co-sleepers and habitual individual sleepers, regarding all assessed parameters, using unpaired two-tailed t-tests or Wilcoxon rank sum tests (in case of lacking normal distribution).

Then, we tested whether there was a latency until partner effects on sleep hygiene would be fully present (i.e., a biphasic relationship between sleeping-arrangement routine and SHI scores). Therefore, we plotted 5-point central moving averages of the sleeping-arrangement duration and the respective SHI scores and conducted a curve-fit analysis. We tested linear, polynomial (up to the 5^th^ order), and square root functions, which then were compared using ANOVAs. Moving averages were calculated to smoothen the data to better explore underlying patterns. Please note, while moving averages are usually used for time series, they have also been applied to pseudo-time-series obtained by retrospective cross-sectional studies^14^.

This explorative analysis of the relationship between co-sleep duration and sleep hygiene lead to a preliminary cut-off value in co-sleep duration that described the phase shift between adaptational period and plateau phase with optimal SHI.

This preliminary cut-off value was then validated in the original dataset. SHI values of four groups (co-sleep above or below the cut-off and individual sleep above or below the cut-off) were compared using pairwise, unpaired two-tailed t-tests. Controlling for multiple testing was done using the method of Benjamini and Hochberg (1995)^15^, which is based on controlling the false discovery rate.

Finally, we tested the ability of bed-sharing to predict sleep hygiene relative to other relevant determinants of health behavior. We did multiple linear regression analysis in co-sleepers with SHI as the dependent variable and phase of co-sleeping routine (latency or plateau), self-control, depressiveness, age, gender, and nationality as independent variables. A stepwise variable selection method was used. The same procedure was done in individual sleepers. Here, stability of the sleeping arrangement (i.e., individual sleep) was used as a predictor variable to test whether a possible effect of a stable sleeping arrangement in co-sleepers would merely be a function of a stable sleep routine or is in fact result of a social influence.

It is of note that even though a traditional view would consider the number of cases per predictor variable (n=6.7) in the regression analyses as low, recent evidence shows that even fewer cases per variable suffice to accurately predict Beta coefficients and R^2^s^16^. All calculations and graphs were done in R (version 3.4.4). Significance was set as *p*<0.05. Data are available upon request.

## RESULTS

### Sample characteristics and group comparisons

When comparing individual sleepers and co-sleepers - irrespective of the continuity of the sleeping arrangement - both groups did not differ significantly in any of the collected variables (all *p*s≥0.117; Table 1).

### Exploring the time-dependent relationship of co-sleep and sleep hygiene in a processed (smoothened) dataset

#### A biphasic relationship between co-sleep routine and sleep hygiene

**Figure 1:**
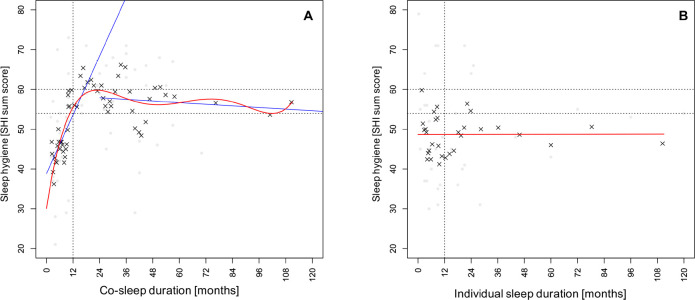
*Temporal relationship between sleeping-arrangement and sleep hygiene.* There is a biphasic relationship between continuity of sleeping arrangement and sleep hygiene in habitual co-sleepers (A) that is missing in habitual individual sleepers (B). A: The relationship can be described as quintic polynomial equation (red curve) or as two linear processes with following correlations: r=0.83, *p*<0.001 (left blue line) and r=-0.17, *p*=0.425 (right blue line). **Notes:** Tests: Spearman correlations; Dataset: processed with simple central moving averages (±2 values, black crosses), light-grey dots represent original data points.

In habitual bed-sharers a quintic polynomial equation with an adjusted R^2^ of 0. 671 (p<0.001; Figure 1A) fitted the dataset best as compared to lower-order polynomial equations (all R^2^ s≤0.645, all Fs≥4.19, all *p*s≤0.046). The graph is characterized by: i) an initial steep incline; ii) a peak at about two years of co-sleep routine; iii) a subsequent plateau in a corridor between SHI scores of 54 and 60 (Figure 1A). The apparent linear downwards tendency in the plateau phase from the time of the maximum is not statistically significant (r=-0.17, *p*=0.425, n=24, Spearman correlations, Figure 1A, right blue line). In contrast, the initial increase represents a very high positive linear correlation (r=0.83, *p*<0.001, n=34, Spearman correlation, Figure 1A, left blue line).

In habitual individual sleepers none of the polynomial fits was superior to a straight nearly horizontal line (all *p*s≥0.732; r=0.04, *p*=0.819, Figure 1B).

#### One year as cut-off value

Before the maximum, the curve enters the corridor between SHI scores of 54 and 60 for the first time slightly before 12 months. This indicates that one year of continuous co-sleep routine with the same partner might be a candidate for a cut-off between the two phases of the biphasic relationship between co-sleep routine and sleep hygiene.

### The one-year cut-off is confirmed in the unprocessed dataset

**Figure 2:**
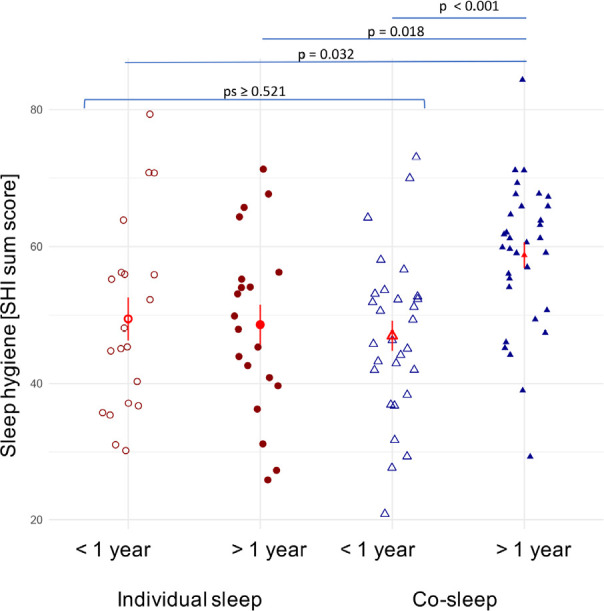
*Sleep hygiene in co-sleepers and individual sleepers above and below the one-year cut-off.* Co-sleepers with more than 1y routine of co-sleeping, have significantly better sleep hygiene than co-sleepers with less than one year of co-sleeping or individual sleepers.

**Notes:** Red dots represent means (± SEM, vertical red lines); Dataset: original, unprocessed data; Tests: pairwise, unpaired two-sided, T-tests; Multiple testing adjustment: Benjamini and Hochberg (1995)^15^.

With a mean (±SD) of 58.8 (± 10.7), co-sleepers above the one-year cut-off scored significantly higher in the SHI as those below the cut-off (47.0±11.8, *p*<0.001; Figure 2) as well as individual sleepers on both sides of the one-year cut-off (48.6±13.1, *p=*0.006 for >1y individual sleep and 49.5±14.0, *p*=0.016, for <1y individual sleep; Figure 2). The latter three groups did not differ significantly from each other (all *p*s≥0.521; Figure 2). Controlling for multiple testing did not render any previously significant *p*-value non-significant (*p*<0.001, *p*=0.018, and *p*=0.032 respectively).

### Predicting sleep hygiene in co-sleepers and individual sleepers

To investigate the effect of other parameters on sleep hygiene, we conducted multiple linear regression analyses in co-sleepers and individual sleepers using a stepwise variable selection. The resulting models were highly significant (both *p*s≤0.001) and explained 26% (co-sleepers) and 48% (individual sleepers) of the variances in sleep hygiene (Table 2). In co-sleepers, the final model contained two predictor variables, namely >1y of co-sleep routine (standardized β=0.421, *p*<0.001) and depressiveness (standardized β=-0.25, *p*=0.029). In individual sleepers, the model also contained two predictor variables one of which was self-control (standardized β=0.616, *p*≤0.001). Albeit being included in the final model, the second predictor (depressiveness) was non-significant (standardized β=-0.19, *p*=0.134). Other predictor variables (i.e., nationality, gender, age) were excluded via the stepwise variable selection process and did not become part of the best models. Details are given in Table 2.

**Table 2:** Multiple linear regression models to determine predictors of sleep hygiene.

Model	SHI type (dependent variable)	Sleeping arrangement (model descriptors)	Predictor variables	Standardized beta coefficient [95% CI, lower, upper]	p-value
**1a)**	**SHI total score**	Co-sleep	Co-sleep >1year	0.42 [0.2, 0.65]	<0.001
		(R^2^=0.257; *p*<0.001)	Depressiveness	-0.25 [-0.48,-0.03]	0.029
1b)		Individual sleep	Self-control	0.62 [0.36, 0.87]	<0.001
		(R^2^=0.481; *p*<0.001)	Depressiveness	-0.19 [-0.44, 0.06]	0.134

**Notes:** Tested predictor variables: sleeping-arrangement continuity, self-control, depressiveness, age, gender, nationality. Variable selection technique: stepwise. Given are adjusted R^2^s. SHI = Sleep hygiene index.

## DISCUSSION

The present study is the first to investigate the effect of bed partners on sleep hygiene with a focus on the temporal dimension and differential factors predicting sleep hygiene in habitual co-sleepers and habitual individual sleepers. We report two major findings: first, a biphasic relationship of continuity of the sleeping arrangement and sleep hygiene in co-sleepers but not in individual sleepers, and second, differential, robust predictors of sleep hygiene in co- and individual sleepers, namely co-sleep routine and self-control.

The first finding complements the only other study dealing with timely aspects of relationships and sleep^5^. This work reports that - after controlling for a plethora of confounders - only long-term relationship stability is associated with better subjective and objective sleep^5^. However, the time periods in question differ. We show a phase transition to a stable plateau phase at approximately one year of continuous co-sleep routine. Troxel et al. (2010)^5^ report on relationship stability across the whole 6-8 years observation period being advantageous as compared to never being partnered or having gained or lost a partner. Since they do not report the time point during the observation period at which the relationship transition took place, it is impossible to infer the trajectory of the relationship between relationship duration and sleep quality in their study. Also, sample differences might be relevant. Troxel et al. (2010)^5^ investigate a multiethnic sample of middle-aged American women whereas we look at rather young predominantly European individuals of both genders. Yet, given that an importance of the temporal factor has been found in such diverse samples indicates that the temporal dimension could be a basic and important determinant of sleep in intimate relationships.

Regarding the second major finding of this study (i.e., determinants of sleep hygiene in co-sleepers and individual sleepers), the fact that bed-sharing of more than one year is associated with better sleep hygiene whereas continuity of individual sleep is not, shows the partner’s capacity to influence sleep hygiene. This is in line with the social-control hypothesis of health behavior^6^. Therefore, it supports the hypothesis that a partner directly influences sleep-related health behavior^1^. The importance of the social factor in predicting sleep hygiene in co-sleepers is moreover stressed by our regression analyses that demonstrate its robustness in face of - amongst others - depressiveness and self-control. This is particularly interesting as the latter both have been reported to impact health behavior^7,11^.

In contrast to the findings in habitual co-sleepers, self-control is the only significant predictor of sleep hygiene in individual sleepers and unaltered continuity of individual-sleep is not. This is an interesting finding for two reasons. First, the ability to exert self-control has been suggested to rely on sleep quality^17^. Combining this finding with ours (i.e., self-control impacts sleep hygiene) forms a positive feedback loop: sleep > self-control > sleep hygiene > sleep. This feedback loop could be clinically relevant since it could represent a perpetuating vicious circle of decreasing sleep quality and self-control. This vicious circle might - according to our results - be more relevant in habitual individual sleepers and might be broken by the social factor in co-sleepers. That could explain reports of better subjective and objective sleep quality in habitually co-sleeping partners^2,5^.

Second, the differential factors determining sleep hygiene in co-sleepers and individual sleepers might represent a starting point for improving CBTI. This could be done by including the partner into CBTI (as suggested by Rogojanski et al. (2013)^1^, and for individual sleepers, self-control training could become more central to the intervention (for review on improving self-control see Duckworth et al. (2018)^18^).

It is of note that the present work it is also limited to some extent. First, it is a cross-sectional study that infers temporal information from combining subjects with different durations of unaltered sleeping arrangement. Therefore, we cannot differentiate whether the co-sleep-duration-dependent aspect is associated with actually improving sleep hygiene over time or whether bad sleep hygiene is more frequent in short-lasting relationships. Second, the durations of unaltered sleeping arrangement are not equally distributed across the sample with an underrepresentation of higher values. Third, despite controlling for common confounders that affect sleep and relationships (age, gender, depressiveness, or nationality), other potential confounders were not controlled for (e.g., life-style habits, personality traits, relationship quality, attachment style^4^, or presence of a medical condition such as snoring and obstructive sleep apnea, or insomnia)^19^. Fourth, we did not ask whether the partner was also participating in the study. In light of the fact that only in one case relationship and co-sleep durations are identical for a male and a female participant (which should be the case if they belonged to the same couple), that limitation seems to be negligible in the present study. Finally, the generalizability of the present work might be limited since our convenience sample is not representative for the general population and focusing on dyads complicates transferability to more complex familial constellations (e.g., including little children).

## CONCLUSION

The present study elucidates the importance of the temporal dimension for sleep hygiene in co-sleepers and shows differential factors predicting sleep hygiene in habitual co-sleepers and individual sleepers, namely co-sleep duration of more than one year and depressiveness, and self-control, respectively.

These findings are relevant for the study of sleep in couples and might provide a rationale and starting point for improving CBTI by making it sensitive to the habitual sleeping arrangement. Nevertheless - given the first of a kind nature of the present work - its results should be treated as suggestive rather than conclusive and should be tested in future works, particularly in longitudinal and interventional studies.
